# MicroRNA-1 induces apoptosis by targeting prothymosin alpha in nasopharyngeal carcinoma cells

**DOI:** 10.1186/1423-0127-18-80

**Published:** 2011-11-07

**Authors:** Cheng-Der Wu, Yuan-Sung Kuo, Han-Chung Wu, Chin-Tarng Lin

**Affiliations:** 1 Institute of Pathology, College of Medicine, National Taiwan University No. 7, Chung-Shan S. Rd, Taipei 10002, Taiwan; 2 Department of Surgery, National Taiwan University Hospital, Taipei, Taiwan No. 7, Chung-Shan S. Rd, Taipei 10002, Taiwan; 3 Institute of Cellular and Organismic Biology, Academia Sinica No. 128, Academia Rd, Section 2, Nankang, Taipei 11529, Taiwan; 4 Department of Pathology, National Taiwan University Hospital, No. 7, Chung-Shan S. Rd, Taipei 10002, Taiwan

**Keywords:** nasopharyngeal carcinoma (NPC), microRNA-1 (miR-1), PTMA (prothymosin alpha, ProTalpha), apoptosis

## Abstract

**Background:**

MiR-1 (microRNA-1) has been used as a positive control in some microRNA experiments. We found that miR-1 transfection of nasopharyngeal carcinoma cells reveals a typical apoptotic process as shown by time-lapse microscopy so we investigated the mechanisms of miR-1 inducing apoptosis.

**Methods:**

To confirm that miR-1 induces apoptosis, we used Annexin V and TUNEL staining and caspase assay. To determine that miR-1 directly targets genes that involve in apoptosis, we analyzed microRNA and pathway databases, and cDNA expression microarrays from miR-1 transfected cells. To demonstrate candidate miR-1 targeted genes, we used qRT-PCR analysis and luciferase reporter vector assays. To assess the miR-1 target gene PTMA (prothymosin alpha, ProTalpha) involves in apoptosis, we used PTMA siRNA to knock down PTMA.

**Results:**

Annexin V and TUNEL staining and caspase assay confirm that miR-1 induces nasopharyngeal carcinoma cell apoptosis. MiR-1 transfection of HeLa, Cal-27, KYSE30 and NPC-TW06 cell lines which express low levels of endogenous miR-1 also induces apoptosis. However, miR-1 transfection of cell lines such as SW620, HepG2, HEK-293T, SAS and PC-13 which express high levels of endogenous miR-1 does not result in apoptosis. MiR-1 directly targets PTMA gene. PTMA siRNA and miR-1 accelerate the apoptotic process in cells treated with apoptosis inducers.

**Conclusions:**

The exogenous expression of miR-1 induces apoptosis in a number of cell lines. This is a model of microRNA-induced cell apoptosis. The PTMA is one of miR-1 target genes which involve in miR-1 inducing apoptosis. The apoptotic inducers including actinomycin D, camptothecin and etoposide are also the chemotherapeutic drugs in clinical cancer therapy and PTMA siRNA can accelerate apoptotic progression in cells treated with those apoptosis inducers. Therefore PTMA siRNA may have potential applications as an adjuvant in cancer chemotherapy.

## Background

MicroRNAs are endogenous non-coding RNAs that are approximately 21 nucleotides long. They are evolutionarily conserved and found in many animals, plants, fungi and viruses. MicroRNAs function as regulators of gene expression and play important roles in biological processes [1,2]. In previous investigation of pathogenic microRNAs in nasopharyngeal carcinoma (NPC) cells, we used miR-1 (microRNA-1) to transfect NPC cells as a positive control. To our surprise, we observed a typical apoptotic response in miR-1 transfected NPC cells with time-lapse microscopy. Negative control microRNA and other microRNAs did not induce cellular apoptosis under identical transfection conditions. Transfection of miR-1 also caused apoptosis in other cancer cell lines such as HeLa, Cal-27 and KYSE30.

Apoptosis is a form of spontaneous cell death characterized by externalization of phosphatidylserine, cell shrinkage, chromatin margination and condensation, nuclear fragmentation, activation of caspases, cellular budding and production of apoptotic bodies [3,4]. Apoptosis is one of the important biological processes for organisms. Many diseases have been associated with abnormal apoptosis; excessive apoptosis has been linked to auto-immunity and insufficient apoptosis plays a large role in cancer transformation.

In the present study, we confirm that miR-1 directly targets PTMA mRNA. PTMA also known as prothymosin-alpha or ProTalpha is a hormone or hormone-like polypeptide precursor first isolated from the rat thymus gland [5]. PTMA functions in transcription regulation and cell proliferation [6,7], and acts as an apoptotic inhibitor by binding to Apaf-1 [8,9]. This study shows that PTMA siRNA and miR-1 accelerate the apoptotic process in cells treated with apoptotic inducers in comparison to the control. Hence the PTMA siRNA and miR-1 may have a potential therapeutic application for cancer therapy.

## Methods

### Cell lines

The following cell lines were used for this study: HeLa, human cervical adenocarcinoma [ATCC CCL-2.2], Cal-27, human tongue carcinoma [ATCC CRL-2095 ], KYSE30, human esophageal carcinoma [JCRB0188 ], HepG2, human hepatocellular carcinoma [ATCC HB-8065 ], PC13, human lung large cell carcinoma [IBL], SW620, human colorectal adenocarcinoma [ATCC CCL-227 ], SAS, human tongue carcinoma [JCRB0260 ], HEK-293T, human transformed embryonal epithelial cell [ATCC CRL-11268 ], NPC-TW01 and NPC-TW06 were human nasopharyngeal carcimoma that were established in our laboratory [10-12]. All cell lines were cultured in DMEM containing 5% fetal calf serum and L-glutamine and incubated in a 10% CO_2 _incubator.

### Transfection of microRNA and siRNA

The microRNAs in this study were purchased from Ambion (Austin, TX, USA); the microRNAs included Pre-miR™ hsa-miR-1 miRNA precursor positive control (Cat# AM17150), Pre-miR™ hsa-miR-486 miRNA precursor (Cat# PM10546), Pre-miR™ hsa-miR-429 miRNA precursor (Cat# PM10221), Pre-miR™ hsa-miR-200a miRNA precursor (Cat# PM10991) and Pre-miR™ miRNA precursor negative control #1 (Cat# AM17110). A total of 1.5 × 10^5 ^cells were seeded in each well of a 6-well culture plate in 1 ml of normal culture medium for microRNA transfection. The microRNA transfection mixture was prepared by diluting 1 μl of 50 μM microRNA in 100 μl of Opti-MEM I reduced serum medium (GIBCO, Gaithersburg, MD, USA). A total of 6 μl of HiPerFect transfection reagent (QIAGEN, Valencia, CA, USA) was added to the diluted microRNA and mixed by vortexing. After incubation for 10 minutes at room temperature, normal culture medium was added to mixture to bring the final volume to 1 ml. The microRNA transfection mixture was added to the well containing cells and mixed gently. A mock-transfected control was prepared by the same process but without the addition of microRNA. Another untransfected control (blank) was also prepared by the same process but without the addition of microRNA and transfection reagent. The culture medium in all experimental groups was changed after 24 hours to the regular medium and transfected again. The siRNA transfection conditions of PTMA Pre-design ChimeraRNAi (Abnova Cat# H00005757-R01, Taipei, Taiwan) and AllStars negative control siRNA (QIAGEN Cat#1027281) were identical to those for the microRNA transfection.

### Apoptosis assay

The morphological features of cell apoptosis were observed by ASTEC CCM-MULTI time-lapse microscopy (ASTEC, Fukuoka, Japan). Phosphatidylserine was stained using an Annexin V FITC assay kit (Serotec, Raleigh, USA). DNA fragments were stained using a DeadEnd Fluorometric TUNEL system (Promega, Madison, WI, USA). Caspase activity was detected by a Caspase-Glo caspase 3/7 assay kit (Promega) and Z-VAD-FMK caspase inhibitor (Promega). All of the experimental processes were performed according to manufacturer instructions.

### Microarray analysis

NPC-TW01 and HeLa cells were transfected with miR-1 and miR-negative control and transfected again after 24 hours. Total RNA was extracted using Trizol (Invitrogen, Gaithersburg, MD, USA) after 42 hours. Total RNA from the miR-1 transfected cells was labeled with Cy5; total RNA from the miR-negative transfected cells was labeled with Cy3. The Cy-labeled RNAs were hybridized to a human whole genome oligo 4 × 44 K microarray (Agilent, Santa Clara, CA, USA). After washing and drying, the microarrays were processed with the Agilent microarray scanner; microarray data were analyzed by the GeneSpring GX 11 and Ingenuity Pathways analysis system. All microarray experiments were performed according to Agilent protocols. The microarray data had been submitted to NCBI GEO (http://www.ncbi.nlm.nih.gov/geo) under the accession numbers GSM706489 and GSM706490.

### Quantitative RT-PCR

For mRNA quantification, total RNA was extracted by Trizol (Invitrogen) and reverse transcribed by Superscript III (Invitrogen). The quantitative real-time polymerase chain reaction (qRT-PCR) experiments were performed using the ABI Power SYBR Green PCR Master Mix with the ABI 7500 Real-Time PCR System (Applied Biosystems, Austin, TX, USA). All of the experimental protocols were performed according to the manufacturer's instructions. qRT-PCR primer sequences are comprehensively listed in table 1. For endogenous miR-1 quantification, microRNA was reverse-transcribed using a TaqMan MicroRNA reverse transcription kit (Applied Biosystems, Cat#4366597) and primers using the TaqMan endogenous controls RNU6B (Applied Biosystems, Cat#4373381) and TaqMan MicroRNA assays hsa-miR-1 (Applied Biosystems, Cat#4373161). Remaining procedures were performed identically to mRNA quantification.

**Table 1:** Primers used for qRT-PCR

Name	Symbol	Entrez Gene ID	Sequences (5' to 3')
C5-QF	C5	NM_001735	CGGTGCTGGAGTTTAATGTCAA
C5-QR	C5	NM_001735	TGGCTGAGAGATGAGTATGCTATTG
CARD8-QF	CARD8	NM_001184900	TTTCTCAGGTGCAGCCTTTG
CARD8-QR	CARD8	NM_001184900	CTCCACCAGCTCCTTCTCAT
FAIM-QF	FAIM	NM_018147	ACAGCGGGTGAGTTTGTAGA
FAIM-QR	FAIM	NM_018147	CTTCCCACTACTGACAGCCT
GRIN2A-QF	GRIN2A	NM_001134408	GACAAGGATCCGACGTCTACCT
GRIN2A-QR	GRIN2A	NM_001134408	GCATGACCGTGGCTTGCT
PTMA-QF	PTMA	NM_002823	CGAAATCACCACCAAGGACTT
PTMA-QR	PTMA	NM_002823	GTCAGCCTCCTGCTCCC
TP63-QF	TP63	NM_001114982	GCAGTTGTGTTGGAGGGATGA
TP63-QR	TP63	NM_001114982	CCCATCTCTGGTTTCCAGAGTAAC
GAPDH-QF	GAPDH	NM_002046	GCACCGTCAAGGCTGAGAA
GAPDH-QR	GAPDH	NM_002046	AGGGATCTCGCTCCTGGAA

### Luciferase reporter assay

DNA fragments of miR-1 binding sites (wild-type and mutant) in the PTMA 3'UTR were synthesized and cloned into the multiple cloning sites (SacI-XbaI) of pmirGLO vector (Promega). All constructions were confirmed by DNA sequencing. The constructions were co-transfected with or without miR-1 into NPC-TW01 cells. Luciferase activity was measured 36 hours after transfection using the Dual-Glo luciferase assay system (Promega) and Orion II microplate luminometer (Berthold, Bad Wildbad, Germany). All experimental protocols were performed according to the manufacturer's instructions. DNA fragment sequences are list in table 2.

**Table 2:** Oligonucleotides used for pmirGLO luciferase reporter vector construction

Name	Sequences(5' to 3')
PTMA#1-wt-se	ACGAGCTCTTGTATTTTTTATTTACATTTTATCTAGAG
PTMA#1-wt-as	CTCTAGATAAAATGTAAATAAAAAATACAAGAGCTCGT
PTMA#1-mut-se	ACGAGCTCTTGTATTTTTTATTATGTAATTATCTAGAG
PTMA#1-mut-as	CTCTAGATAATTACATAATAAAAAATACAAGAGCTCGT

PTMA#2-wt-se	ACGAGCTCAAACAATCTTATTCCGAGCATTCCATCTAGAG

PTMA#2-wt-as	CTCTAGATGGAATGCTCGGAATAAGATTGTTTGAGCTCGT

PTMA#2-mut-se	ACGAGCTCAAACAATCTTATTCCGAGGTAAGGATCTAGAG

PTMA#2-mut-as	CTCTAGATCCTTACCTCGGAATAAGATTGTTTGAGCTCGT

pmirGLO-sequencing-primer	TGAAGAGCCTGATCAAATAC

### Treatment of apoptotic inducers

For assessing the apoptotic effects of PTMA siRNA or miR-1, a total of 5 × 10^3 ^NPC-TW01 cells were seeded in each well of a 96-well culture plate and were transfected with PTMA siRNA, miR-1 or control as mentioned above. After transfection for 48 hours, actinomycin D apoptotic inducer was added to the culture media to a final concentration of 5 μM (or camptothecin 10 μM, or etoposide 200 μM). Cells were subsequently observed by time-lapse microscopy (ASTEC, Fukuoka, Japan). Another 96 well plates (which experimental conditions were identical to that plate for time-lapse microscopy observation) were performed MTT assay for quantitating cell viability.

## Results

### MiR-1 induces NPC cell apoptosis

**Figure 1:**
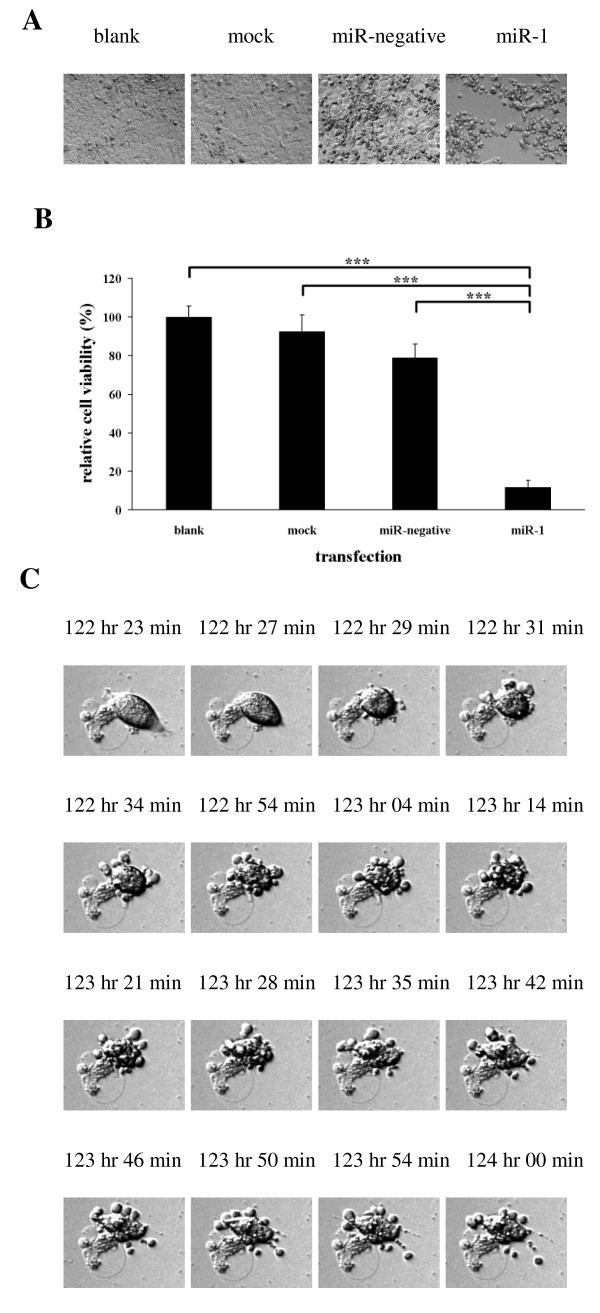
**Transfection of miR-1 into NPC-TW01 cells induces apoptosis**. From left to right: untransfected NPC-TW01 cells (blank), transfection reagent only (mock), transfection reagent and miR-negative control (miR-negative), and transfection reagent and miR-1 (miR-1). Photos were taken by time-lapse microscopy 150 hours after transfection. (B) MTT assay after transfection with miR-1 for 150 hours. Most miR-1 transfected cells were dead (12% cell viability), whereas only a few control transfected died. (***Comparison between two groups as indicated, p < 0.001. Error bars correspond to mean ± SD) (C) The process of apoptotic body budding was visualized by time-lapse microscopy in miR-1 transfected cells. The time above each photograph represents the specific time that NPC-TW01 cells were transfected with miR-1.

**Figure 2:**
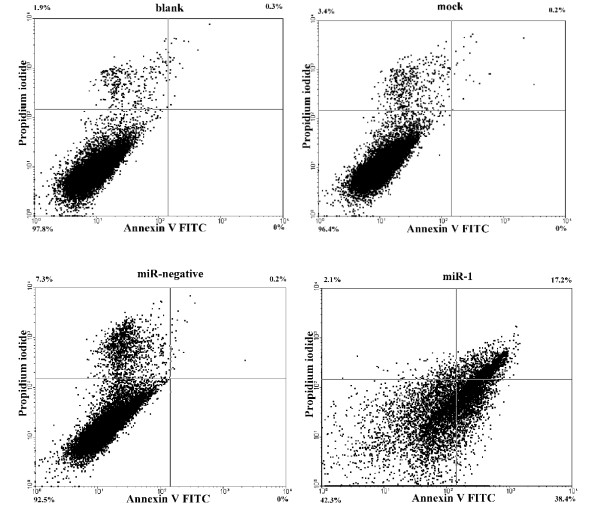
**Annexin V analysis of apoptotic cells**. Untransfected NPC-TW01 cells (blank), transfection reagent only (mock), transfection reagent and miR-negative control (miR-negative), and transfection reagent and miR-1 (miR-1) separately. After transfection for 90 hours, the cells were stained by annexin V and propidium iodide and detected by flow cytometry. The percentage of cells stained in each quadrant is listed in the corner of each quadrant. The upper left quadrants represent oncosis cells, lower left quadrants represent normal cells, upper right quadrants represent necrotic cells (late apoptotic or oncotic cells) and lower right quadrants represent early apoptotic cells.

**Figure 3:**
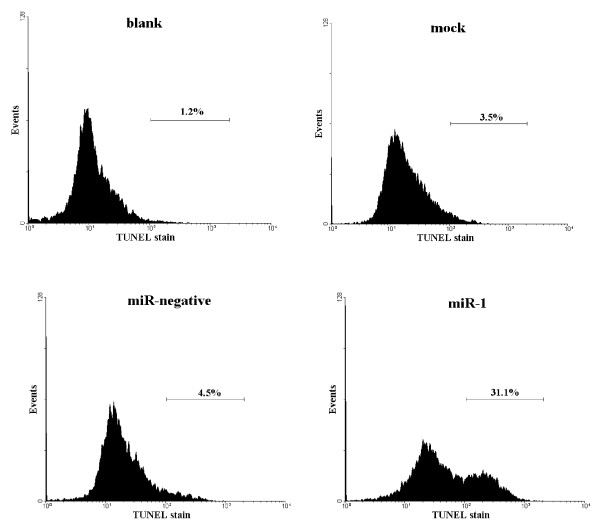
**TUNEL assay of the fragmented DNA from apoptotic cells**. Clockwise from top left: untransfected NPC-TW01 cells (blank), transfection reagent only (mock), transfection reagent and miR-1 (miR-1) and transfection reagent and miR-negative control (miR-negative). After transfection for 120 hours, the cells were subjected to TUNEL assay. TUNEL-stained cells were quantitated by flow cytometry. The number in each panel is the percentage of TUNEL-positive cells.

NPC-TW01 cells were transfected with either miR-1 or control and observed by time-lapse microscopy. The miR-1 transfected cells appeared to undergo apoptosis as only 12% of cells survived after transfection for 150 hours; the majority of the cells transfected with control survived (Figure 1A, 1B). The miR-1 transfected cells displayed typical apoptotic morphology, including cell shrinkage and budding of apoptotic bodies (Figure 1C). Further analysis by Annexin V staining (Figure 2) and TUNEL assay (Figure 3) revealed that there were more apoptotic cells in sample transfected with miR-1 than sample transfected with control. We used the Promega Caspase-Glo caspase 3/7 assay kit to measure caspase 3/7 activities in the NPC-TW01 cells transfected with miR-1. 48 hours after transfection, for miR-1 transfected cells demonstrated a 4.6-fold increase in caspase 3/7 activities compared to the untransfected cells (p < 0.01). A subset of the NPC-TW01 cells was treated 50 μM of the broad-spectrum Promega Z-VAD-FMK caspase inhibitor for 1 hour before transfection with miR-1. After 120 hours of miR-1 transfection, a MTT assay determined the viability of the cells treated with caspase inhibitor was 2.5-fold higher compared to the cells not treated with caspase inhibitor (p < 0.01). MiR-1 transfection of NPC-TW01 cells induced apoptosis, whereas NPC-TW01 cells transfected with other microRNAs (miR-200a, miR-429 and miR-486) did not induce cellular apoptotic morphology as observed by time-lapse microscopy (data not shown). Transfection of miR-1 into HeLa, Cal-27, KYSE30, and NPC-TW06 cells generated apoptotic bodies but these changes in morphology did not occur after transfection of SW620, HepG2, SAS, HEK-293T and PC-13 cell (data not shown). The endogenous expression of miR-1 in different cell lines was analyzed by qRT-PCR. The endogenous miR-1 expression level in NPC-TW01 cells was used as a standard for comparison. Cells with low endogenous miR-1 expression levels (Cal-27 = 0.01-fold, KYSE30 = 0.13-fold, HeLa = 0.04-fold, and NPC-TW01 = 1-fold) could be induced to undergo apoptosis via miR-1 transfection; cells with high endogenous miR-1 expression levels (SW620 = 155-fold, HEK-293T = 86.9-fold, HepG2 = 2.6-fold, SAS = 3-fold, and PC-13 = 9.7-fold) could not be induced to undergo apoptosis by miR-1 transfection.

### MiR-1 directly targets PTMA

**Figure 4:**
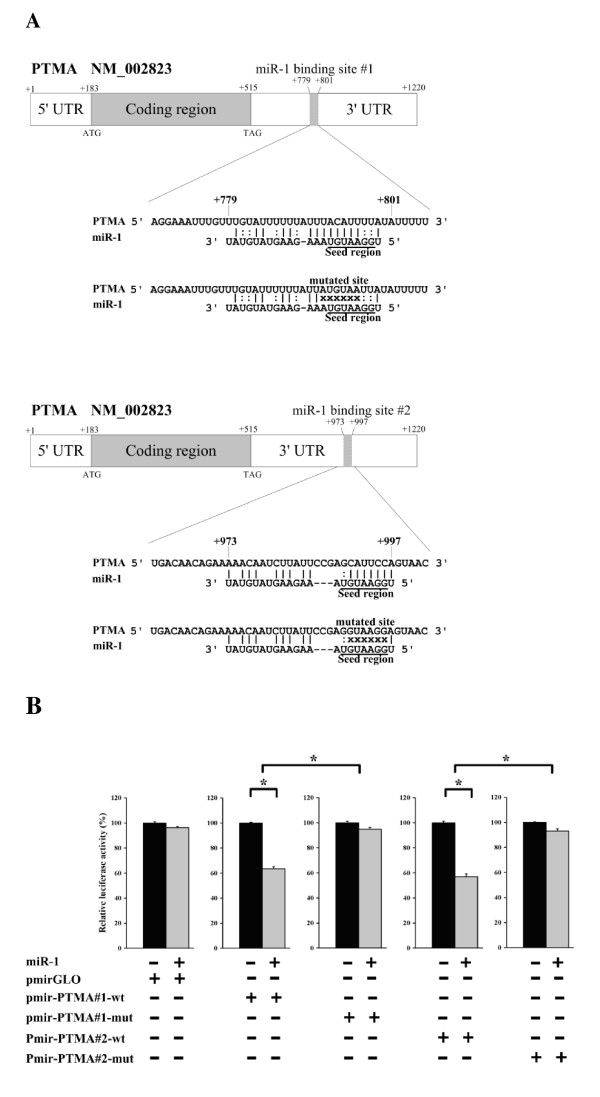
**Luciferase assay demonstrates that miR-1 directly targets PTMA 3' UTR**. (A) Schematic representation of the miR-1 targeting sequences that complement with the 3' UTR of PTMA mRNA. The PTMA 3'UTR (+779~801 and +973 ~ +997 nt, miR-1 predictive binding site) were cloned into pmirGLO luciferase vector to generate pmir-PTMA#1-wt and pmir-PTMA#2-wt. The miR-1 seed region complementary sequences were mutated and named as pmir-PTMA#1-mut and pmir-PTMA#2-mut. (B) NPC-TW01 cells were transfected with miR-1 or without any microRNA. NPC-TW01 cells were then transfected with pmirGLO empty vector, pmir-PTMA#1-wt, pmir-PTMA#2-wt, pmir-PTMA#1-mut or pmir-PTMA#2-mut. Luciferase assays were performed after 36 hours of transfection. The results indicate that miR-1 directly binds to the PTMA mRNA 3'UTR and impairs PTMA expression in NPC-TW01 cells. (*Comparison between two groups as indicated, p < 0.05. Error bars correspond to mean ± SD)

To investigate the mechanisms of miR-1-induced apoptosis, we performed a cDNA expression microarray analysis. We then screened microRNA database and pathway database to identify the candidate miR-1-targeted genes involved in apoptosis. During a 42-hour transfection of NPC-TW01 and HeLa cells with miR-1 or miR-negative control total RNA was isolated from the cells and hybridized to an Agilent human whole genome oligo 4 × 44 K microarray. Using GeneSpring GX 11 (microarray analysis software) to compare the mRNA expression profiles between miR-1 and miR-negative transfected cells, a total of 2473 transcripts were found to be down regulated over two-fold in NPC-TW01 cells. A total of 2063 transcripts were found to be down regulated more than two-fold in HeLa cells. To rule out cell-line-specific expression transcripts, we combined the NPC-TW01 and HeLa cells down regulated transcripts and obtained 279 transcripts. The 279 transcripts were compared to the miR-1 predictive targeting genes from the miRTarBase (http://mirtarbase.mbc.nctu.edu.tw) and miRecord database (http://mirecords.biolead.org). A total of 86 transcripts were identified as targets of miR-1. The 86 transcripts were further analyzed with the Ingenuity pathways analysis system (http://www.ingenuity.com). From Ingenuity pathways database analysis; the down regulation of six transcripts (C5, CARD8, FAIM, GRIN2A, PTMA, and TP63) could increase apoptosis (Table 3). To verify if the six transcripts are regulated by miR-1 in other cell lines, nine different cell lines were transfected with or without miR-1; mRNA levels of the six candidate genes were quantitated by qRT-PCR. The PTMA (prothymosin alpha, ProTalpha) transcript was down regulated in all nine cell lines during miR-1 transfection (Table 4). Therefore, we focused on PTMA and performed a luciferase reporter vector assay to determine if miR-1 can directly regulate PTMA expression. The miR-1 binding site (predict by miRanda) was inserted into the reporter vector and co-transfected with or without miR-1 into NPC-TW01 cells. The luciferase activity of miR-1 transfected cells was significantly decreased compared to that of cells not transfected with miR-1. However, if the reporter vector bore the mutated miR-1 binding site and was co-transfected with or without miR-1, the luciferase activity was not significantly distinguishable while comparing miR-1 transfected and untransfected cells (Figure 4). All data indicate that miR-1 impairs PTMA mRNA expression by directly binding to the PTMA mRNA 3'-UTR in NPC-TW01 cells.

**Table 3:** Candidate miR-1 targeted genes involved in apoptosis

Symbol	Entrez Gene Name	Entrez Gene ID	Fold change in NPC-TW01	Fold change in HeLa
C5	complement component 5	727	-2.96	-2.36
CARD8	caspase recruitment domain family, member 8	22900	-4.74	-2.72
FAIM	Fas apoptotic inhibitory molecule	55179	-4.28	-2.54
GRIN2A	glutamate receptor, ionotropic, N-methyl D-aspartate 2A	2903	-2.54	-2.31
PTMA	prothymosin, alpha	5757	-6.62	-2.76
TP63	tumor protein p63 (TP73L)	8626	-2.37	-2.33

NPC-TW01 and HeLa cells were transfected with miR-1 and their total RNA was hybridized to microarrays. Transcripts that were down regulated more than two-fold were screened as target genes of miR-1 using the miRTarBas and miRecord databases. The target genes were then screened for involvement in apoptosis using the Ingenuity pathways database.

**Table 4:** qRT-PCR confirming miR-1 down regulates genes in different cell lines

Symbol\cell	NPC-TW01	NPC-TW06	HEK-293T	Cal-27	HeLa	HepG2	KYSE30	SAS	SW620
C5	0.16	0.71	0.19	0.44	0.21	0.24	0.32	0.11	1.53
CARD8	0.44	0.94	0.17	0.63	1.04	0.31	0.04	0.58	0.71
FAIM	0.22	0.88	0.21	0.62	0.80	0.29	0.44	0.21	1.32
CRIN2A	1.05	0.19	0.09	1.36	0.43	0.24	0.80	1.51	0.31
PTMA	0.29	0.28	0.28	0.26	0.45	0.45	0.42	0.11	0.62
TP63	0.69	1.12	0.07	2.45	0.92	0.27	1.00	0.88	0.43

Nine cell lines were transfected with or without miR-1; total RNA was isolated from these cells after 24 hours of transfection. mRNA from the six candidate genes were quantitated by qRT-PCR, normalized to GAPDH mRNA, and compared to levels in the untransfected samples. PTMA mRNA is down regulated in all nine miR-1 transfected cell lines.

### PTMA siRNA assists cells progression to apoptosis

**Figure 5:**
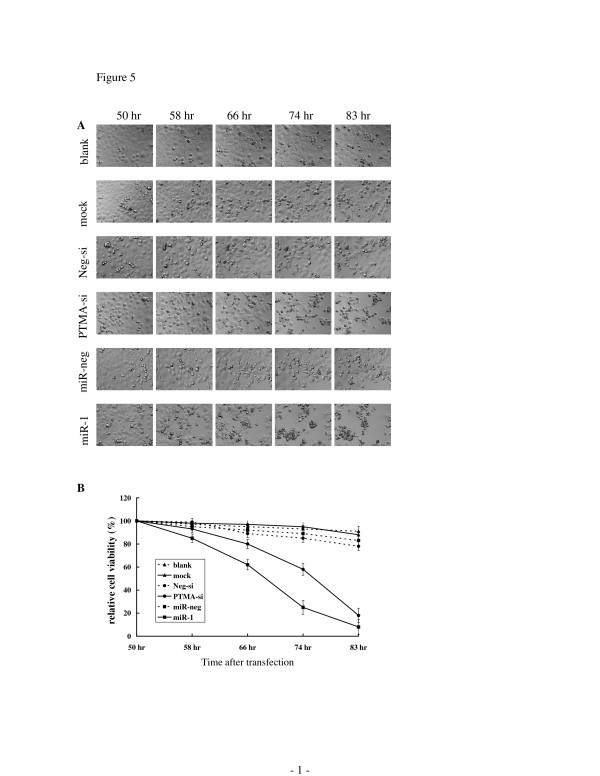
**PTMA siRNA and miR-1 accelerate the apoptotic process in cells were treated with actinomycin D**. From top to bottom: untransfected NPC-TW01 cells (blank), transfection reagent only (mock), transfection reagent and negative control siRNA (Neg-si), transfection reagent and PTMA siRNA (PTMA-si), transfection reagent and miR-negative control (miR-neg) and transfection reagent and miR-1 (miR-1). Actinomycin D apoptotic inducer was added to culture media to a final concentration of 5 μM after 48 hours of transfection. Cells were subsequently observed by time-lapse microscopy (A). The time above each photograph represents the specific time that cells were transfected with microRNA, siRNA or control. (B) Cell viability was quantitated by MTT assay. The experimental conditions of the MTT assay were identical to those during time-lapse microscopy observation. Transfection of PTMA siRNA or miR-1 accelerated the apoptotic process after the NPC-TW01 cells were treated with actinomycin D apoptotic inducer.

Propelling by evidence that miR-1 induces apoptosis and directly knocks down PTMA mRNA expression; we investigated the role of PTMA in apoptosis by transfecting NPC-TW01 cells with PTMA siRNA. There was no difference in apoptotic morphology between PTMA siRNA and control transfected cells as observed by time-lapse microscopy (data not shown). In fact, transfection of NPC-TW01 cells with PTMA siRNA knocked down PTMA mRNA expression 0.027-fold (p <0.01) relative to untransfected control cells; mRNA expression was analyzed by qRT-PCR after transfecting for 24 hours. The results showed that knocking down PTMA mRNA expression alone does not induce apoptosis. We propose that the apoptosis mechanism involves the down regulation of apoptotic inhibitors and up regulation of apoptotic inducers. Because PTMA has previously been reported as an apoptotic inhibitor [8,9], we performed another siRNA experiment in which NPC-TW01 cells were transfected with PTMA siRNA or miR-1. After transfection for 48 hours, apoptotic inducer actinomycin D was added to the culture media. Cell viability was evaluated by MTT assay and cell morphology was observed by time-lapse microscopy. As shown in figure 5, apoptosis appeared earlier in the PTMA siRNA and miR-1 transfected cells treated with actinomycin D relative to control transfected cells. Treating cells with other apoptotic inducers such as camptothecin and etoposide produced the same results as those of actinomycin D (data not shown). These results indicate that knock down of PTMA expression alone does not induce apoptosis. However, PTMA siRNA and miR-1 transfections in conjunction with the addition of apoptotic inducers accelerate the apoptotic process in NPC-TW01 cells.

## Discussion

MiR-1 can be used as a positive control and for optimizing transfection conditions in microRNA experiments (e.g., Ambion Pre-miR miRNA Starter Kit, QIAGEN Syn-hsa-miR-1 miScript miRNA mimic positive control). In this study, we noticed that transfection of NPC-TW01 and other cancer cell lines with exogenous miR-1 induce cell apoptosis. Endogenous miR-1 expression may be linked to apoptosis; for example, myocardial infarction increases miR-1 expression and induces apoptosis in rat H9C2 myoblast cells [13,14]. MiR-1 expression also increases and apoptosis is induced when rat cardiomyoctes are incubated with H_2_O_2 _[15] and treated with high glucose [16,17]. The mir-1-1 gene is methylated and has reduced expression in human hepatocellular carcinoma cell lines compared to normal liver cells. When the mir-1-1 gene is hypomethylated, it re-expresses miR-1 and can induce apoptosis [18]. In mouse myoblast cells, the mutation of MyoD transcription factors down regulates miR-1 expression and decreases cell apoptosis [19]. MiR-1 expression is down regulated in human lung cancer tissues and cell lines in comparison to levels in normal human lungs. MiR-1 affects lung cancer cells in response to anti-cancer drugs [20]. MiR-1 is down regulated in human bladder cancer [21] and head-neck squamous cell carcinoma (HNSCC) cells [22]. Microarray gene expression profiles of miR-1 transfected cells revealed 17 miR-1 targeted candidate genes, they included PTMA. The authors demonstrated that miR-1 directly targets TAGLN2 and acts as a tumor suppressive microRNA [21,22]. All reports in existing literature indicate that miR-1 plays an important role in apoptosis and cancer pathogenesis. In this study, we show that cell lines with low levels of endogenous miR-1 expression were apoptosis inducible via transfection with miR-1. Cell lines with high endogenous miR-1 expression were not apoptosis inducible by transfection with miR-1. Further testing with more cell lines is needed to obtain a definitive conclusion. MiR-1 directly down regulates PTMA mRNA levels and induces apoptosis. We used PTMA siRNA to down regulate PTMA mRNA levels and observed whether PTMA siRNA could induce apoptosis. Knocking down PTMA expression alone did not induce apoptosis but accelerated apoptotic progression in cells treated with apoptosis inducers. These results are in correlation with previous reports that PTMA is an apoptotic inhibitor and has anti-apoptotic properties [9,23,24]. The anti-apoptotic mechanism is that PTMA binds to Apaf-1 then prevents Apaf-1/cytochrome C apoptosome formation. Without apoptosome formation, pro-caspase-9 cannot be activated to induce the caspase cascades that lead to apoptosis [8,9]. MiR-1 induces apoptosis and targets PTMA. PTMA is an apoptotic inhibitor, and knock down of PTMA expression levels is not enough to induce cell apoptosis. Therefore, miR-1-induced apoptosis is likely to alter the expression of other pro-apoptotic proteins. We found an up regulation of some pro-apoptotic proteins (HTRA, SMAC, p53, TNF-R1, TNF-R2, TRAILR-1, TNF-alpha, TNF-beta) expression in miR-1 transfected cells (unpublished data). MicroRNA and pathway database analysis reveals that miR-1 indirectly regulates those pro-apoptotic protein expressions. We will verify the pathway network between miR-1 and those pro-apoptotic proteins in the near future.

## Conclusions

We have demonstrated that exogenous expression of miR-1 can induce apoptosis in some cell lines. This is a model of microRNA-induced cell apoptosis. PTMA is one of miR-1 target genes that involve in apoptosis. PTMA siRNA and miR-1 can accelerate the apoptotic process in NPC-TW01 cells when cells are treated with apoptotic inducers. The apoptotic inducers: actinomycin D, camptothecin and etoposide are also as chemotherapy drugs in clinical cancer therapy so miR-1 and PTMA siRNA may have potential applications in assisting cancer therapy.

## Competing interests

The authors declare that they have no competing interests.

## Authors' contributions

CDW designed experiments. CDW and YSK performed major experiments and data analysis. HCW and CTL interpreted the data. CDW and CTL drafted manuscript. All authors read and approved the final manuscript.
